# Efficacy of Angiotensin-Converting Enzyme Inhibitors in Coronary Microvascular Dysfunction: A Systematic Review and Meta-Analysis of Randomized Clinical Trials

**DOI:** 10.7759/cureus.52684

**Published:** 2024-01-21

**Authors:** Mohamed R Abouzid, Samar Eldahtoury, Shorouk M Elshafei, Sunita Devi, Amr Saleh, Sadaf Esteghamati, Ibrahim Kamel

**Affiliations:** 1 Internal Medicine, Baptist Hospitals of Southeast Texas, Beaumont, USA; 2 Internal Medicine, Mansoura University, Mansoura, EGY; 3 Internal Medicine, University of La Verne, La Verne, USA; 4 Internal Medicine, Carney Hospital, Boston, USA

**Keywords:** meta-analysis, coronary flow reserve, microvascular angina, ace inhibitors, coronary microvascular dysfunction

## Abstract

Coronary microvascular dysfunction (CMD) is becoming increasingly recognized as an important contributor to the development of ischemic heart diseases. Without obstructive coronary artery disease, the physiological function of the coronary microcirculation can be altered by structural, functional, and molecular factors, leading to myocardial ischemia. CMD can significantly impact the quality of life and prognosis and imposes a huge financial burden on healthcare systems and people. This meta-analysis aims to investigate the efficacy of angiotensin-converting enzyme inhibitors (ACEIs) for treating CMD.

A systematic literature review identified randomized controlled trials (RCTs) comparing ACEIs with placebo in CMD patients. Review Manager, 5.3 for Windows, was utilized. Using the Mantel-Haenszel (M-H) method, improvement in coronary flow reserve (CFR) and systolic blood pressure events was pooled as mean difference (MD) in a meta-analysis model with a fixed effect model, whereas the number of chest pain episodes was pooled as MD with a random effect model. Five randomized controlled trials involving 209 patients were included in the analysis. The analysis demonstrated a statistically significant improvement in CFR in the ACEIs group compared to the placebo group (MD -0.3, 95% CI -0.61 to 0.01, P = 0.05). However, there was no significant difference in the number of chest pain episodes between the ACEIs and placebo groups (MD 1.79, 95% CI -3.99 to 7.58, P = 0.54). Similarly, no significant difference in blood pressure change was observed between the two groups (MD 4.02, 95% CI -3.25 to 11.28, P = 0.28).

In conclusion, the appropriate treatment for CMD is a source of contention because adequate data is lacking. Our findings suggest that ACEIs may have a positive effect on improving CFR in patients with microvascular angina. However, ACEIs did not demonstrate a significant impact on the number of chest pain episodes or systolic blood pressure in this patient population. Further research, including RCTs with larger sample sizes and longer follow-up durations, is warranted to provide more conclusive evidence on the role of ACEIs in CMD management.

## Introduction and background

The primary focus of research and investigation in the field of angina pectoris has predominantly been on the many diseases and pathologies that impact the epicardial coronary arteries. The importance of coronary microcirculation in maintaining sufficient blood supply to the heart muscle has long been recognized, but the significant negative health effects of coronary microvascular dysfunction (CMD) have been increasingly evident [1,2].

Angina without a hemodynamically significant coronary obstruction is a paradox regularly encountered by physicians in the course of their daily medical practice. The extensive utilization of imaging modalities has facilitated the accurate characterization of coronary anatomy through techniques like coronary angiography, coronary computed tomography, or echocardiography. As a result, these modalities have enabled the identification of anginal equivalents and myocardial ischemia, even in cases where there is no evidence of obstructive coronary artery disease (CAD). This condition is referred to by various names, including CMD, cardiac syndrome X, microvascular angina, or ischemic heart disease without obstructed coronary arteries (INOCA). However, because CMD contributes to myocardial ischemia in many of these patients, the concept of microvascular angina may be expanded to include patients with obstructive coronary artery disease and individuals with angina after coronary revascularization or heart transplantation.

CMD is becoming more widely recognized as a serious cardiac illness that can cause signs and symptoms of myocardial ischemia in a variety of clinical scenarios. CMD is also frequently responsible for angina in people with cardiomyopathy and heart valve disease, as well as Takotsubo syndrome and myocardial infarction with no obstructive coronary artery disease. The malfunction is typically found in the coronary microcirculation, with a channel diameter of less than 500 micrometers. Impaired coronary microvasculature vasodilation, microvasculature spasm, endothelial dysfunction, vascular smooth muscle dysfunction, and extravascular compressive pressures have all been identified as possible major pathophysiologic mechanisms for this type of ischemic heart disease [3,4]. The exact underlying mechanisms are numerous, frequently overlap, and remain poorly understood. Other conditions include chronic inflammation, oxidative stress, abnormal cardiac adrenergic stimulation, insulin resistance, dysregulated pain perception, estrogen insufficiency, and hereditary predispositions that have also been documented [5,6]. Anginal symptoms in the absence of obstructive epicardial coronary artery disease or myocardial disease, but the presence of cardiovascular risk factors is a frequent and frequently underdiagnosed clinical manifestation of CMD [3,5]. There is still a scarcity of data on the efficacy of pharmacological therapies for CMD.

This medical condition, which has an impact on individuals of both genders, can have a substantial influence on their overall well-being and future prognosis. It also poses a considerable economic strain on healthcare systems and individuals. In recent times, there have been notable progressions in the diagnosis of myocardial ischemia, along with the utilization of various tests to explore the functional and structural factors contributing to reduced coronary flow reserve and microvascular spasms. As a result, there has been an enhanced ability to identify more instances of microvascular angina in routine clinical settings [1].

Despite improved diagnosis, CMD remains a management problem for involved healthcare providers [7,8]. Despite treatment with standard antianginal medicines such as beta-blockers, calcium channel blockers (CCBs), or long-acting nitrates, a considerable proportion of CMD patients may have persistent symptoms and a poor quality of life [9-13]. As a result, numerous novel medicines with various mechanisms of action have recently been investigated for symptomatic treatment in this patient population. Although antihypertensives are not meant for CMD, recent research has shown that angiotensin-converting enzyme inhibitors (ACEIs) or angiotensin receptor blockers (ARBs) may be effective in improving coronary reserve flow (CFR). Furthermore, data on the efficacy of CMD medicines is limited. The majority of studies on this subject produce inconclusive results. According to the current European Society of Cardiology position document on CMD, medications such as ACEIs, statins, and beta-blockers may be used to treat CMD [14]. Several reviews indicate that exercise, risk factor control, and medications such as ACEIs, ARBs, CCBs, nicorandil, and ranolazine may be effective treatments for CMD [15,16].

In individuals with coronary artery disease, ACEIs reduce sympathetic coronary vasoconstriction and can ameliorate endothelial dysfunction. Based on these findings, this systematic review and meta-analysis were carried out to explore the clinical and statistical relevance of ACEIs in treating CMD.

## Review

Methods

Literature Search

The literature search strategy for this meta-analysis aimed to identify relevant studies on the use of ACEIs in treating CMD. Key concepts included ACEIs, CMD, and treatment, including various synonyms and keywords for ACEIs, CMD, and treatment, which allowed for a comprehensive exploration of relevant literature, minimizing the risk of missing key studies. The search was conducted on reputable academic databases such as PubMed, Embase, and Cochrane Library, as well as clinical trial registries and relevant medical journals. Filters and limits, such as publication date ranges and study types, were applied as needed. The search strategy was designed to effectively capture a broad spectrum of research articles and clinical trials to inform the meta-analysis. This systematic approach to literature retrieval serves as the foundation for the subsequent stages of study selection, data extraction, and analysis.

Eligibility Criteria

We included randomized controlled trials (RCTs) that meet the following PICO criteria: (1) population (P): patients with microvascular angina diagnosed with the stress test, echocardiography, or based on clinical picture; (2) intervention (I): ACEIs regardless of name, dosage, route, and duration of administration; (3) comparison (C): placebo; (4) outcomes (O): the primary outcome is the change in CFR. Our secondary outcomes are the number of chest pain episodes and the decrease in systolic blood pressure. Animal studies, in vitro investigations (tissue and culture studies), observational studies (cross-sectional, case-control studies, cohort studies, case series, and case reports), single-arm clinical trials, in vitro investigations (tissue and culture studies), book chapters, editorials, press articles, conference abstracts, and non-English language studies were excluded.

Study Selection and Data Extraction

This meta-analysis was conducted according to PRISMA guidelines. Three investigators (S.E. S.E. & S.D.) independently performed electronic database searches on PubMed, EMBASE, and ClinicalTrials.gov (inception through December 2022) for RCTs meeting our inclusion criteria using the prespecified keywords and MeSH terms “ACE Inhibitors AND Microvascular Angina” OR “ACE inhibitors and Cardiac Syndrome X” OR “Enalapril and Microvascular angina” OR "ACE inhibitors AND coronary microvascular dysfunction" OR "ACE inhibitors AND INOCA". The pertinent references were also thoroughly sought. Our meta-analysis included all RCTs that investigated the efficacy of ACEIs for treating microvascular angina.

Risk of Bias and Quality Assessment

Three reviewers (S.E., S.E., and S.D.) assessed the risk of bias in randomized trials using The Cochrane Collaboration's tool. The included studies were separately evaluated for their risk of bias. The study took into account various factors that could introduce bias, including random sequence generation (which may lead to selection bias), allocation concealment (which may also lead to selection bias), blinding of participants and personnel (which may introduce performance bias), blinding of outcome assessment (which may introduce detection bias), incomplete outcome data (which may introduce attrition bias), selective reporting (which may introduce reporting bias), and other potential sources of bias. Conflicts were effectively resolved through the process of engaging in constructive dialogue and deliberation. The quality of evidence assessment in this study was conducted by two reviewers (I.K. and S.E.) using the Grading of Recommendations Assessment, Development, and Evaluation (GRADE) Working Group suggestion. The study considered many factors, such as inconsistency, imprecision, indirectness, publication bias, and bias risk. The justification, documentation, and inclusion of our results on the quality of evidence were evident in the reporting of each outcome. An additional reviewer resolved any disagreements.

Assessment of Heterogeneity

Heterogeneity was assessed by the utilization of visual examination of the forest plots, as well as the application of I-square (I2) and Chi-square testing. The Chi-square test is employed to assess the presence of significant heterogeneity in the effect size, whereas the I-square test quantifies the extent of heterogeneity. The measurement and interpretation of heterogeneity were conducted using the recommendations outlined in Chapter 9 of the Cochrane Handbook of Systematic Reviews and Meta-analysis. According to this guideline, a significance level (alpha) of less than 0.1 is deemed indicative of substantial heterogeneity for the Chi-square test. The I-square test is interpreted as follows: when the value falls between 0% and 40%, it may not be regarded significant; when it ranges from 30% to 60%, it may indicate moderate heterogeneity; and when it falls between 50% and 90%, it may suggest considerable heterogeneity. Applying the random effects model was warranted in cases with a notable degree of heterogeneity. In the remaining instances, the fixed effect model was employed.

Data Synthesis

Review Manager, 5.3 for Windows, was utilized. Using the Mantel-Haenszel (M-H) method, improvement in CFR and systolic blood pressure events was pooled as mean difference (MD) in a meta-analysis model with a fixed effect model, whereas the number of chest pain episodes was pooled as MD with a random effect model. Due to the heterogeneity of some included studies, the random effect model was employed, whereas the fixed effect model was utilized under the premise that the included studies were homogenous and equivalent in terms of research design, quality, and measurements of treatment effect.

Publication Bias

According to Egger and colleagues, assessing publication bias in less than ten combined studies is unreliable. Therefore, Egger's test for funnel plot asymmetry could not be utilized to establish whether or not our study contained publication bias [17].

Results

Search Results and Study Selection

**Figure 1:**
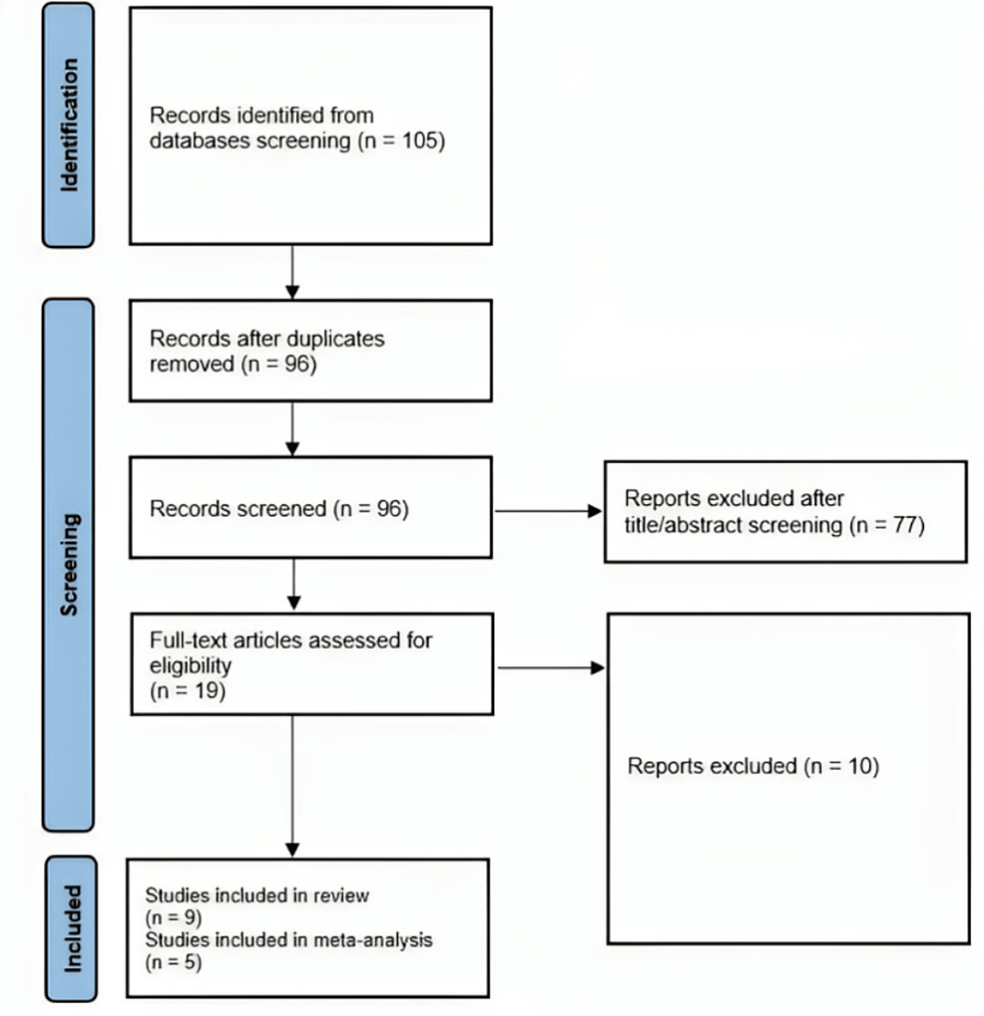
PRISMA Flow Chart

One hundred five articles were collected by searching electronic databases, mainly PubMed. After title and abstract screening, 86 records were excluded, leaving 19 articles for full-text screening. Ten articles were excluded after the full-text screening. Our systematic review included nine articles and five articles with 158 patients were eligible for our final analysis [2,18-25]. Figure 1 shows the selection process in a PRISMA flow diagram. The summary of the included studies with their main results is illustrated in Table 1.

**Table 1:** Summary of the Included Studies Pauly D et al. 2011 [2], Kaski J et al. 1994 [18], Michelsen et al. 2018 [19], De Gregorio et al. 2021 [20], Nalbantgil et al. 1998 [21], Pizzi et al. 2003 [22], Mangiacapra et al. 2013 [23], Chen et al. 2002 [24], and Motz et al. 1996 [25]. ACEI: angiotensin-converting enzyme inhibitors; CFR: coronary flow reserve; CMD: Coronary microvascular dysfunction; PCI: percutaneous coronary intervention

Study	Design	ACEI used	Follow-up period	Result
Pauly D et al. 2011 [2]	Double-blinded randomized controlled trial	Quinapril	16 weeks	The primary efficacy criterion assessed in the study was CFR, whereas the secondary parameter evaluated was the absence of angina symptoms. There was a notable improvement in CFR among the therapy group. Both quinapril and CFR demonstrate a considerable improvement in secondary outcomes.
Kaski J et al. 1994 [18]	Randomized, single-blind, crossover, placebo-controlled trial	Enalapril	2 weeks of treatment with enalapril and after 2 weeks of placebo administration	All placebo patients exhibited exercise-induced ST segment depression and angina, while 6 enalapril patients had good results (4 with angina). Enalapril extended exercise duration, time to 1mm ST segment change, and ST depression, all of which were significant. Peak exercise heart rate and blood pressure were similar.
Michelsen et al. 2018 [19]	Randomized double-blinded, superiority Trial	Ramipril	24±6 weeks	Follow-up data is available for 55 patients. CFR improvement was not statistically different between treatment and placebo groups (p= 0.63). The groups' symptoms improved similarly. The systolic and diastolic function did not change.
De Gregorio et al. 2021 [20]	Pilot study	Perindopril	6 months	There was no substantial improvement observed in myocardial blood flow among individuals with hypertrophic cardiomyopathy and CMD who had a 6-month treatment course with perindopril.
Nalbantgil et al. 1998 [21]	Randomized double-blind crossover placebo-controlled trial	Cilazapril	3 weeks, followed by 3 weeks of the other therapy	The therapeutic efficacy of Cilazapril was observed in cases of CMD through its ability to modulate coronary tone at the microcirculatory level.
Pizzi et al. 2003 [22]	randomized, prospective, single-blind, placebo-controlled fashion	Ramipril	6 months	The use of atorvastatin with ramipril over a period of six months has been found to enhance endothelial function and improve the overall quality of life in individuals with CMD.
Mangiacapra et al. 2013 [23]	Prospective, randomized, double-blind, controlled study	Enalaprilat	10 minutes after administration and after PCI	The administration of intracoronary enalaprilat has been shown to enhance the functionality of the coronary microvasculature and provide protection to the myocardium against harm resulting from procedures in patients diagnosed with coronary artery disease who are having PCI
Chen et al. 2002 [24]	Double-blinded randomized trial	Enalapril	1.5 months	Long-term enalapril treatment improved exercise performance and CFR
Motz et al. 1996 [25]	Double-blinded randomized trial	Enalapril	11 to 13 months	The administration of enalapril over an extended period of time resulted in enhanced coronary microcirculation in individuals with hypertensive heart disease.

Quality of Included Studies

**Figure 2:**
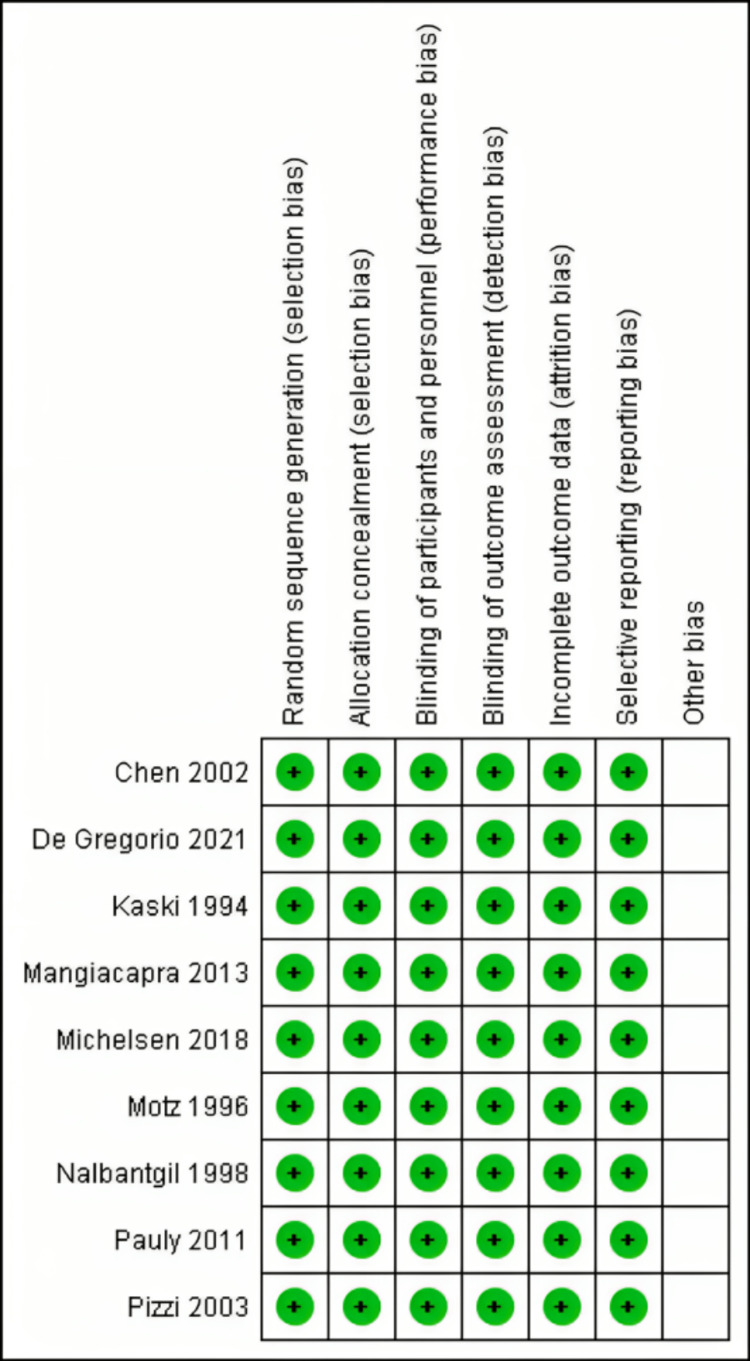
Risk of Bias Summary Pauly D et al. 2011 [2], Kaski J et al. 1994 [18], Michelsen et al. 2018 [19], De Gregorio et al. 2021 [20], Nalbantgil et al. 1998 [21], Pizzi et al. 2003 [22], Mangiacapra et al. 2013 [23], Chen et al. 2002 [24], and Motz et al. 1996 [25]

**Figure 3:**
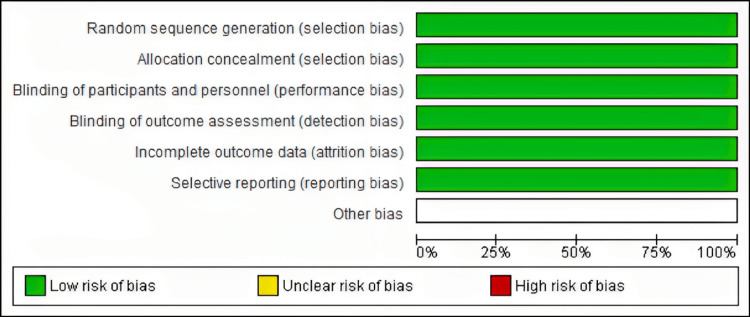
Risk of Bias Graph

Using the Cochrane risk of bias assessment technique, the quality of the included studies ranged from low to high quality. Figure 2-3 summarize the quality assessment domains of the included studies.

Efficacy Analysis (From Baseline to Endpoint)

**Figure 4:**
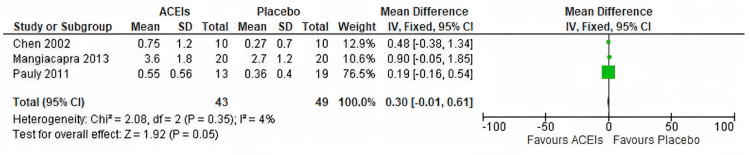
Forest plot of mean difference of change in CFR Pauly D et al. 2011 [2], Mangiacapra et al. 2013 [23], and Chen et al. 2002 [24]. CFR: coronary flow reserve

Coronary flow reserve: The pooled analysis of the three studies assessing the effect of ACEIs on CFR yielded a mean difference (MD) of 0.30 with a 95% confidence interval (CI) of (-0.01-0.61) and a p-value of 0.05, as shown in Figure 4. The results suggest a trend towards improvement in CFR with the use of ACEIs, although the effect did not reach statistical significance. It is important to note that a p-value of 0.05 indicates a marginal level of statistical significance, and further research with larger sample sizes may be needed to confirm these findings. The pooled studies were homogeneous (P = 0.35; I^2^ = 4 %).

**Figure 5:**
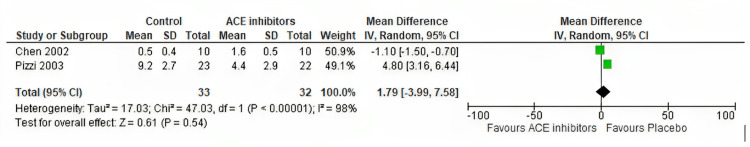
Forest Plot of Number of Chest Pain Episodes Pizzi et al. 2003 [22] and Chen et al. 2002 [24]

Number of chest pain episodes: Two studies contributed to this outcome. There is no statistically significant difference in the number of chest pain episodes between the ACEIs group and placebo group [MD 1.79, 95 % CI (-3.99-7.58), P = 0.54; Figure 5]; the pooled studies were heterogeneous (P = < 0.00001; I^2^ = 98 %).

**Figure 6:**
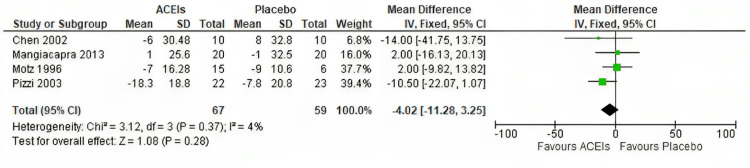
Forest plot of change in systolic blood pressure Pizzi et al. 2003 [22], Mangiacapra et al. 2013 [23], Chen et al. 2002 [24], and Motz et al. 1996 [25]

Systolic blood pressure: Four studies contributed to this outcome. There is no statistically significant difference in systolic blood pressure between the ACEIs group and placebo group [MD - 4.02, 95 % CI (-11.28-3.25), P = 0.28; Figure 6]; the pooled studies were homogeneous (P = 0.37; I^2^ = 4 %).

Discussion

Harvey Kemp coined cardiac syndrome X in 1973 [26]. It is characterized by either classic or atypical anginal chest pain and the absence of substantial coronary vascular abnormalities on angiography. This syndrome is also known as CMD, microvascular angina, and chest pain with normal coronary arteries. It is considered a form of ischemic heart disease, with perimenopausal and postmenopausal women being the most susceptible [27].

The presence of symptoms of angina best characterizes microvascular angina due to microvascular dysfunction and the presence of nonobstructive CAD. It is diagnosed if the following criteria are present: (1) symptoms suggesting myocardial ischemia; (2) objective evidence of myocardial ischemia determined as per the current protocol e.g., rest/stress electrocardiography; (3) absence of a significant obstruction in the coronary vasculature (<50% obstruction in the coronary arteries and/or fractional flow reserve (FFR) > 0.80); (4) confirmed reduction in coronary blood flow reserve and/or inducible microvascular spasm.

Patients with CMD have risk factors with those suffering from epicardial CAD. The toxic prooxidant effects of cigarette smoking involve coronary microcirculation in addition to the epicardial vessels [28]. It has been demonstrated that hyperlipidemia causes CMD in people with normal coronary arteries [29]. Patients with hypertension are predisposed to develop CMD [30]. Multiple studies have demonstrated that people with diabetes mellitus (DM) develop CMD, which may be an early indicator of atherosclerosis and epicardial coronary artery disease (CAD) in this population [31]. Compared to the general population (8%), individuals with established CMD have a 30% increased likelihood of presenting with metabolic comorbid disorders [19].CMD has been linked to inflammation, such as in patients with systemic lupus erythematosus and rheumatoid arthritis [32].

Contrary to epicardial coronary arteries, cardiac microcirculation cannot be clearly visualized using coronary angiography [33]. An endomyocardial biopsy can be used to visualize coronary microvessels; however, it is highly invasive and cannot be used to evaluate the microcirculation's functionality [34]. Several metrics based on measuring blood flow through the coronary circulation have been utilized to characterize coronary microvasculature function. The definition of CFR is the maximum increase in coronary flow that can be achieved from the resting state to peak coronary vasodilation [35]. Since resistance to flow in the coronary circulation is primarily determined by the microvasculature, CFR can serve as a proxy for microvascular performance. The gold standard for clinically evaluating microvascular function is currently CFR by positron-emission tomography (PET) or cardiovascular magnetic resonance imaging (CMR) [33]. It is still unknown which CFR threshold should be used to establish CMD, particularly as it has been demonstrated that the CFR of healthy individuals varies with age and sex [36]. An early PET research proposed a cutoff value 2.5 as the normal range [37].

Nonetheless, several recent prognostic studies on individuals with and without CAD have revealed that CFR thresholds of 1.5 to 2.6 have predictive value [10,13,38-41]. There are some invasive methods for measuring CFR, such as thermodilution techniques and intracoronary Doppler ultrasonography. These methods assess blood flow through a single epicardial artery, examining a single coronary distribution. Physicians must not simply depend on the angiographic appearance of epicardial coronary artery stenosis when assessing patients with myocardial ischemia. Acquiring a deep understanding of the physiological aspects of coronary blood vessels and the existing techniques for studying them can enhance the accuracy of diagnosing and predicting outcomes in invasive evaluations of coronary circulation. This information can also aid in making better clinical decisions [7,33].

As its mechanisms are incompletely understood, the treatment of patients suspected of having CMD is difficult and intricate. In some people, CMD may be the underlying main pathology, whereas in others, it may be a subsequent clinical feature of another pathology. There are currently no established treatments for CMD. Variable patient selection due to the lack of a consistent, standardized diagnosis, inappropriate study designs, small sample sizes, and insufficient evidence of clinical improvement have hampered the results of available therapy studies [1]. In terms of symptom management, the ESC guidelines published in 2014 propose beta-blockers as first-line medication and calcium channel blockers if the beta-blockers are ineffective or intolerable in affected patients [1,14,18,35]. Intriguingly, there is evidence that long-acting nitrates are useless or perhaps harmful in the treatment of coronary microvascular disorders [7]. In this regard, ACEIs have recently been pushed as a potential treatment option for CMD by blunting the sympathetic effects on coronary vasoconstriction, improving endothelial dysfunction, and preventing myocardial remodeling [15,18].

Few data exist about the effectiveness of ACEIs in the treatment of CMD. The available trials had small sample sizes and conflicting findings; hence, there is insufficient evidence to justify using ACEIs for CMD. According to Michelsen et al., ramipril did not significantly improve coronary microvascular function in individuals, as measured by CFR, compared to placebo. Furthermore, no effect of ramipril medication on the burden of symptoms or indices of left ventricular systolic or diastolic function was seen compared to placebo. Ramipril did not affect systolic or diastolic blood pressure [19]. In the CARAPaCE trial, treatment with perindopril for 6 months did not significantly increase coronary blood flow in patients with hypertrophic cardiomyopathy and CMD [20].

Kaski et al. reported that enalapril improves exercise-induced angina and ST segment depression in patients with CMD by implying that ACEIs attenuate sympathetic coronary vasoconstriction. It was found that 40% of patients had a negative exercise test result, and enalapril significantly increased exercise duration and time to 1 mm of ST segment depression in the treatment group compared to placebo [18].

The precise methods by which ACEIs enhance coronary microcirculation in CMD patients are still a matter of debate. As potential underlying causes, researchers have hypothesized impaired coronary microvasculature vasodilation, microvasculature spasm, aberrant cardiac adrenergic activation, endothelial dysfunction, vascular smooth muscle dysfunction, and extravascular compressive stresses [5,6,21]. The resistance of coronary vessels was found to be affected by several factors, including angiotensin-converting enzyme (ACE) and local angiotensin II (AT-II) levels [42-45]. According to Pizzi et al., six months of treatment with atorvastatin plus ramipril improved endothelial function measured by flow-dependent endothelium-mediated dilation and quality of life measured by exercise ability and symptoms of daily life in CMD patients [22]. Pauly et al. also reported that ACEIs treatment in women with CMD was associated with significant improvement in CFR and angina symptom frequency measured by the Seattle Angina Questionnaire (SAQ) at a 16-week follow-up period [2]. These medications may reduce oxidative stress in the coronary circulation. Statins and ACEIs were prescribed for CMD because of their high antioxidant and anti-inflammatory characteristics. Inhibition of the Renin-Angiotensin system is associated with decreased free radical concentration and oxidative stress. Additionally, it increases coronary flow reserve through bradykinin-mediated, nitric oxide-dependent pathways [21,22].

The effects of ACEIs on microvascular circulation and peri-procedural outcomes in patients with stable angina undergoing percutaneous coronary intervention (PCI) were explained by Mangiacapra et al. in the ProMicro study [23]. In addition to improving CFR, intracoronary enalaprilat was demonstrated to minimize PCI-related myocardial damage. In ST-segment elevation myocardial infarction patients undergoing PCI, intracoronary enalaprilat enhanced coronary blood flow after reopening the affected coronary artery. The protective role of enalaprilat was explained by a decrease in neurohormonal and inflammatory mediators, such as norepinephrine and endothelin 1, as well as an increase in the availability of vasodilatory mediators including bradykinin and nitric oxide [23].

The findings of our analysis provide preliminary evidence that ACEIs may have a potential benefit in improving CFR in patients with CMD. The observed trend towards improvement in CFR suggests that ACEIs could play a role in dilating the coronary microvasculature and enhancing blood flow to the myocardium. However, the lack of statistical significance highlights the need for further research to establish the efficacy of ACEIs in this context.

Limitations

We acknowledge that our meta-analysis has some limitations. First, the limited sample size and short follow-up period affected our analysis and the statistical significance of the findings and hindered our ability to conduct adequate meta-regression analyses to identify potential correlations between the summary effects and the study-level data. Second, it is essential to note that a substantial proportion of individuals participating in the meta-analyzed trials did not exhibit classic angina. Third, the cross-over design of some studies and differences in follow-up durations between studies can be prone to period effects, resulting in differences in an intervention's efficacy. In the included research, the CFR was evaluated using several methodologies. Fifth, the evaluated variability between studies was substantial. Due to insufficient data, the results must be evaluated with extreme caution, and it is impossible to draw definitive conclusions. Sixth, the proportion of concomitant anti-ischemic drugs differed among the included trials, making it harder to assign favorable outcomes to ACEIs than other anti-ischemic agents, which hinders the meta-analysis significantly. Seventh, because ACEIs are primarily epicardial vasodilators, they may not efficiently produce alterations in coronary flow reserve associated with microvascular spasm. Lastly, there can be possible inconsistency in the patients included due to differences in the definition of CMD among studies. However, to our knowledge, this is one of the first systematic reviews and meta-analyses that provides a comprehensive evaluation of the use of ACEIs in patients with CMD.

## Conclusions

CMD is a condition with a high prevalence, a bad prognosis, and negative clinical implications. Emerging noninvasive tools like CMR and PET can accurately evaluate coronary microvascular function. A standard definition of CMD has not yet been defined; however, it has been demonstrated that patients with CMD with or without epicardial CAD have a poor prognosis, including an increase in cardiac death, nonfatal MI, and frequent hospitalizations.

The optimal treatment for CMD is unknown due to the paucity of evidence supporting current medications for CMD. ACEIs appear to be associated with improvements in the CRF and several SAQ domains, including angina frequency, exercise capacity, and quality of life. The lack of statistical significance, however, can be attributed to the small sample size, heterogeneity, duration, and variability of the follow-up periods. Clearly, sufficiently powered large-size randomized clinical trials examining the long-term safety and efficacy of ACEIs in patients with CMD are required.
